# Enhancing college students’ AI literacy through generative AI use: a mixed-methods investigation

**DOI:** 10.3389/fpsyg.2026.1728785

**Published:** 2026-03-09

**Authors:** Jingsheng Wang, Bing Bai, Qi An

**Affiliations:** 1 School of Journalism and Communication, Northwest University, Xi’an, China; 2 School of Marxism, University of Chinese Academy of Social Sciences, Beijing, China; 3 Qi Yue School of Media, Cangzhou Normal University, Cangzhou, China

**Keywords:** AI literacy, college students, generative artificial intelligence, mixed-method approach, network literacy, structural equation modeling

## Abstract

The rapid integration of generative artificial intelligence (GenAI) into higher education has created a paradoxical landscape for college students: while technological advancements offer unprecedented convenience, they simultaneously exacerbate the knowledge-practice gap in AI Literacy cultivation. Traditional educational frameworks struggle to address the dynamic interplay between AI-mediated learning environments, ethical dilemmas, and competency development, leaving a critical theoretical and practical void in literacy cultivation models. To bridge this gap, this study pioneered an exploratory sequential mixed-methods design, combining qualitative interviews (*n* = 30) and quantitative surveys (*n* = 590, response rate 98.33%) to unravel the mechanisms through which GenAI use enhances students’ AI Literacy. Qualitative analysis revealed a spiral-ascending literacy construction model characterized by iterative cycles of cognition-practice-evaluation, wherein 82% of participants demonstrated critical awareness of algorithmic biases and privacy risks. Quantitative results further validated a novel theoretical framework, showing that the social environment indirectly drives application practice via perceived impressions (path coefficient = 0.294, *p* < 0.001), with group needs fully mediating this relationship (*p* = 0.439 for the direct path). Structural equation modeling also identified key pathways linking perceived ease of use (*β* = 0.477) and technological expectations (*β* = 0.284) to behavioral adoption and future-oriented literacy. These findings challenge linear literacy models by emphasizing ecological dynamics and recursive learning processes, offering actionable insights for designing AI-integrated curricula and policies. Collectively, this research underscores the necessity of multi-dimensional interventions, combining cognitive scaffolding, ethical education, and skill training, to transform passive AI utilization into active literacy cultivation in the digital age.

## 1 Introduction

Generative artificial intelligence, a landmark technology of the Fourth Industrial Revolution, is reshaping social structures and individual cognitive paradigms with unparalleled depth and breadth. The “Report on the Development and Application of Generative Artificial Intelligence (2024)” released by the China Internet Network Information Center in 2024 shows that the scale of Generative AI users in China has exceeded 230 million, marking its entry into a new stage of large-scale application from technological exploration.

In the realm of education, this transformation is particularly profound. It presents both a historic opportunity to empower personalized learning and unleash creative productivity in higher education and has also sparked extensive concerns regarding academic integrity, cognitive inertia, data privacy, and ethical violations (Mohammadreza et al., 2024). Against this backdrop, the public urgently needs to acquire corresponding new competencies to master technology rather than be dominated by it. For college students, who are the future pillars of national construction, the cultivation and development of their intelligent competencies are not only crucial for their personal career development and quality of life but also closely intertwined with the nation’s technological innovation capabilities and digital competitiveness (Wang et al., 2025).

AI Literacy (AI Literacy), as a multi-dimensional construct, has transcended its early technical domain, which was mainly applicable to professional technicians, and has evolved into a core competency for citizens in the digital age (Kit et al., 2021). Nevertheless, there is a notable “knowledge-practice gap” in the cultivation of college students’ AI Literacy within the current higher education system. Although students can quickly embrace and utilize Generative AI tools to complete academic tasks, their in-depth understanding of the working principles of AI, critical evaluation of generated content, ethical decision-making capabilities in complex scenarios, and innovative thinking in human-machine collaboration are generally lagging (Černý, 2024).

This lag in the development of competencies, in sharp contrast to the rapid spread of technological applications, renders it a key research topic of both theoretical significance and practical urgency to explore how college students can effectively enhance their AI Literacy through the use of Generative AI.

Existing research has several limitations. Firstly, most studies predominantly focus on depicting the static components of AI Literacy or the technological acceptance of AI use, lacking in-depth analysis of the underlying mechanisms through which “usage behaviors” are dynamically translated into “literacy improvement” (Salhab and Aboushi, 2025). Secondly, in terms of research methodologies, quantitative surveys struggle to capture the intricate contexts and subjective experiences of students’ AI use, while purely qualitative research has difficulty in inferring generalizable patterns. Moreover, the existing literature has not comprehensively integrated multi-dimensional factors such as technology, environment, psychology, and behavior to construct a comprehensive model for explaining the development of literacy (Lu et al., 2024).

Therefore, this study poses the central question: How do the usage behaviors of college students regarding generative artificial intelligence influence and enhance their AI Literacy? This study adopts an exploratory sequential mixed-methods design with the following aims: (1) Through qualitative interviews, delve deep into the authentic experiences, cognitive processes, and literacy challenges encountered by college students when using Generative AI; (2) Based on qualitative findings and existing theories, establish a theoretical model elucidating the relationship between usage behaviors and AI Literacy; (3) Develop and validate scales, and through large-scale questionnaire surveys and structural equation modeling, quantitatively examine the pathways and magnitudes of the impacts of factors such as usage characteristics, psychological cognitions, and environmental support on each dimension of AI Literacy. This study not only endeavors to uncover the formation mechanisms of college students’ AI Literacy but also aims to provide empirical evidence and theoretical guidance for universities to design effective literacy cultivation intervention strategies.

## 2 Literature review

### 2.1 The lag in research on AI literacy

The research trajectory of AI Literacy has prominently exhibited a coexistence of “technology-driven” trends and “theoretical lags.” As early as the 1970s, Agre (1972) introduced the term “artificial intelligence literacy”. Nevertheless, its connotation was narrowly circumscribed at that time as the skills requisite for professional technicians. Over the subsequent decades, progress in related research was sluggish. It was not until the recent exponential proliferation of technologies such as generative AI that AI Literacy was propelled to the forefront of academic research and policy implementation. This delay in the initiation of research has directly contributed to the underdevelopment of the fundamental theoretical framework.

Although the academic community has reached a consensus that AI Literacy constitutes a multi-dimensional construct, there persists a wide array of divergences regarding its specific constituent dimensions, a manifestation of the nascent stage of theoretical development. In the early days, three-dimensional frameworks were predominant. For example, Wong et al. (2020) put forward “AI concepts, AI applications, AI ethics”, and Kim et al. (2021) formulated a model of “AI knowledge, AI skills, AI attitudes”. With the deepening of research, more intricate frameworks have emerged. For instance, Yang et al. (2022) devised a four-dimensional framework within the STEM context, encompassing core concepts, technical practices, interdisciplinary thinking, and ethical attitudes. Zheng et al. (2021) further extended this to a five-dimensional framework incorporating intelligent knowledge, intelligent ability, intelligent thinking, intelligent application, and intelligent attitude. The evolution of these frameworks signals a shift among researchers from the simplistic knowledge—skill dichotomy towards a more holistic perspective of literacy that encompasses ways of thinking and value judgments. However, the proliferation of frameworks has also given rise to challenges in ensuring consistency in concept operationalization, thereby impeding subsequent measurement and comparative research.

An exploration of the essence of AI Literacy is fundamental to understanding its formation mechanism. Some scholars have posited that the essence of AI Literacy lies in the process of “human technologization,” namely, the process through which individuals enhance their inherent capabilities by interacting with technology (Han, 2025). Within this process, emotions (including attitudes, ethics, and a sense of responsibility) and thinking (such as computational thinking, design thinking, and systems thinking) are of equal significance to knowledge itself (Wang et al., 2019). Regrettably, current educational practices often place disproportionate emphasis on knowledge dissemination and tool operation, thereby overlooking the cultivation of the emotional and cognitive dimensions (Gokce et al., 2024).

In the realm of the evaluation system, the development of comprehensive and actionable metrics represents both a current research challenge and a frontier area. Zhong et al. (2024) endeavored to establish an evaluation index spanning the three dimensions of knowledge, emotion, and thinking. In the knowledge dimension, it encompasses aspects such as the understanding and experience of generative AI, system evaluation, and optimization. The emotional dimension encompasses considerations regarding the relationship between AI and humans, ethical and moral aspects, and social norms. The thinking dimension underscores the importance of engineering design thinking (Bocong, 2018) and computational thinking (Zhong et al., 2016), among others. However, the translation of these metrics into reliable and effective tools that can be universally applied across diverse cultural contexts and disciplinary domains remains a formidable challenge. For example, Gokce et al. (2024) employed an artificial intelligence literacy scale that differentiated dimensions such as critical evaluation, practical application, and technical understanding. Their research revealed that students scored notably low in the technical understanding dimension. Conversely, Hobeika et al. (2024) conducted cross-cultural validation of an Arabic version of the artificial intelligence literacy scale, thereby affirming the stability of its four-factor structure. These studies have undoubtedly advanced the development of measurement tools. Nevertheless, the lack of standardization and the proliferation of diverse tools remain significant impediments, rendering direct comparison of research findings arduous and hindering the cumulative growth of knowledge.

While frameworks are proliferating, a comprehensive understanding of AI Literacy must also grapple with the documented drawbacks and limitations of the technologies themselves. A systematic review by Younis et al. (2023) on educational robots and natural language processing, while acknowledging their potential, also synthesized significant practical challenges and risks, such as technical limitations, high implementation costs, and concerns over data privacy. This highlights that the current literature often advances normative competency frameworks without sufficiently grounding them in the systemic obstacles and unintended consequences of the very technologies in use.

### 2.2 Theoretical dialogues and extensions

This study bridges critical gaps in extant theories. While building upon the Unified Theory of Acceptance and Use of Technology (UTAUT) (Venkatesh et al., 2003), it introduces an ecological perspective by incorporating social environment variables. This extension addresses UTAUT’s limited attention to contextual influences, particularly in educational settings (Venkatesh et al., 2003).

Furthermore, the research engages with the Technology Acceptance Model (TAM) by interrogating the mediating role of perceived usefulness and ease of use. Unlike classical TAM, which focuses on individual adoption, this study examines how collective needs and social ecosystems shape literacy development (Davis, 1989).

However, this convenience may come at a cost. Research indicates that students’ preference for AI over human assistance is not purely based on efficacy. For instance, Peng and Wan found that students with higher social anxiety and those facing less complex problems showed a stronger preference for AI teaching assistants, suggesting that reliance on AI can sometimes be driven by psychological comfort rather than pedagogical optimization, potentially reinforcing avoidance of challenging human interaction and deep cognitive engagement (Peng and Wan, 2024).

### 2.3 The urgency of enhancing college students’ AI literacy

The lag in research on AI Literacy stands in sharp contrast to the extreme urgency of enhancing college students’ AI Literacy. This urgency emanates from the profound transformations within the technological society, the distinctive vulnerability and potential of the college student demographic, and the decisive influence of literacy itself on both individual and social development.

The proliferation of generative AI is fundamentally reconfiguring the human-machine relationship. Peng (2022) posits that generative AI represents an “alien” form of intelligence, inherently differing from humans in aspects such as life trajectories and corporeal experiences. This divergence creates an intangible barrier in human-machine communication. Sun, (2023) further underscores that the “inhumanity” of generative AI is more elemental than its “humanity”. Consequently, the traditional concept of human-machine collaboration, characterized by the cooperation of independent entities, is evolving towards a novel relational paradigm of “human-machine overlap and symbiotic integration”. In this new paradigm, college students must acquire corresponding new competencies. Peng (2023) contends that AI Literacy is the evolutionary direction of future competencies, encompassing algorithmic literacy, human-machine collaborative literacy, human-machine communicative literacy, and personal rights literacy, among others. Regrettably, current college students are ill-prepared in these respects. Confronted with the deluge of AI-generated information, they are susceptible to “fear of the unknown” and “intermittent disorientation,” urgently necessitating systematic competency cultivation to navigate this intricate new landscape.

A substantial body of empirical research has unearthed grave issues in college students’ AI Literacy, underscoring the pressing need for intervention. Zhou et al. (2024) discovered in their investigation of Chinese college students that practical experience is pivotal in elevating AI literacy levels. However, students’ positive attitudes and interests have not been fully translated into high-level competency capabilities. Cross-national comparative research has unveiled deeper-seated problems. Mansoor et al. (2024) surveyed college students in Saudi Arabia, Egypt, India, and Malaysia and found that nationality, scientific specialization, and degree level are key determinants of significant disparities in AI literacy levels. Malaysian students, in particular, outperformed their counterparts from other countries, highlighting the decisive role of the social and cultural environment, as well as the educational system, in competency cultivation.

Even more disconcerting are the cascading consequences of insufficient literacy. Firstly, it directly impacts academic achievements and higher-order thinking. Lu et al. (2024) through research based on the 3P model, found that college students’ AI literacy significantly influences their higher-order thinking skills via the mediating effects of behavioral engagement and peer interaction. Secondly, it bears directly on future employability. Ming et al. (2025) demonstrated a clear positive correlation between college students’ perceived AI literacy and their employability. Chun et al. (2025) further corroborated that AI literacy not only directly fosters academic success but also exerts an indirect influence by enhancing self-efficacy and creativity. Conversely, a dearth of literacy not only precludes students from reaping the benefits of technology but also exposes them to the risk of being marginalized in the labor market.

In response to these challenges, both the academic and practical arenas have initiated explorations into diverse cultivation pathways. Shi and Mao (2024) noted in their systematic review that while there is a wealth of competency frameworks and educational research findings applicable to K12 and college student populations, domestic AI literacy education programs and methodologies are still in the nascent stage of development and require further exploration. Some studies have commenced investigations into specific influencing factors and mechanisms. Kong et al. (2025) found that motivational commitment affects AI literacy through the mediating role of self-efficacy, and this relationship exhibits gender disparities. Ke et al. (2025) integrated AI literacy with the UTAUT model and determined that social influence, performance expectancy, and effort expectancy mediate the relationship between AI literacy and the intention to use.

At the disciplinary application level, Zhang (2025) developed a competency framework encompassing technical cognition, practical operation, critical thinking, and creativity for music majors, yielding positive practical outcomes. In the realm of medical education, Song et al. (2025) conducted a quasi-experimental study demonstrating that a generative AI interaction strategy grounded in outcome-based education theory effectively enhances the higher-order thinking and AI literacy of undergraduate nursing students. These studies offer invaluable insights for the cultivation of competencies across different disciplines and scenarios.

Nonetheless, current cultivation practices are beset with numerous challenges. Pan and Zhu (2024) cautioned that the integration of generative AI in college ideological and political courses may give rise to risks such as the erosion of subjectivity, the marginalization of teachers’ discursive power, and threats to ideological security. These issues stem from factors such as the inadequate AI Literacy of educational agents and the opacity of algorithms. Li et al. (2025) illuminated the “digital divide” in generative AI literacy, revealing that students from “Double First-Class” universities significantly outperform their peers from non-“Double First-Class” institutions in multiple aspects, including technical proficiency and critical evaluation. This indicates that inequalities in AI Literacy may exacerbate existing educational disparities.

International frameworks offer valuable guidance. UNESCO’s competency matrix emphasizes ethical reasoning and socio-technical collaboration, resonating with this study’s focus on human-AI synergies. Similarly, Parviainen et al. (2024) advocated for democratized AI education, aligning with our call for multi-dimensional interventions.

In conclusion, the existing literature vividly portrays a landscape where theoretical research on AI Literacy lags behind technological advancements, and the pressing imperative for competency cultivation is met with an unclear path forward. Systematically and empirically exploring how college students utilize generative AI in real-life contexts and identifying the key factors that mediate the transformation of their usage behaviors into higher-order competencies is of paramount significance in bridging the substantial gap between “theoretical frameworks” and “effective cultivation strategies”. Against this backdrop, this study endeavors to penetrate this “black box” through a mixed-method approach, aiming to provide a comprehensive evidence base for addressing the complexities of cultivating college students’ AI Literacy. In extending these models, this study argues for a more ecological perspective. This aligns with broader calls in the field to examine AI in education beyond instrumental adoption. As Cheng et al. (2020) editorialized, research on artificial intelligence and deep learning must move beyond technical performance and grapple with its wider socio-ethical, pedagogical, and ecological implications within educational contexts. Our incorporation of the social environment variable and the focus on literacy as a dynamic construction respond directly to this need for a more situated and holistic investigation.

## 3 Methodology

### 3.1 Research method

This study aims to systematically explore the internal mechanisms and influencing pathways through which college students utilize generative artificial intelligence (GenAI) to enhance their AI Literacy. To delve deeply into this intricate process, an exploratory sequential mixed-methods design is employed. This study adopts an exploratory sequential mixed-methods design (Creswell and Clark, 2017), integrating qualitative interviews and quantitative surveys to achieve theoretical depth and empirical generalizability. This design is particularly suitable for investigating emergent phenomena like AI literacy, where theoretical frameworks are underdeveloped (Teddlie and Tashakkori, 2009). This design initially focuses on qualitative research to thoroughly explore the phenomenon and construct a theoretical model. Subsequently, quantitative research is carried out to test the model with a large sample and verify its general applicability (Lee, 2019). This approach effectively integrates the depth of qualitative analysis and the breadth of quantitative validation, enabling a comprehensive and multi-level response to the research questions.

Firstly, a qualitative research design is adopted in this study. Primary data is collected through semi-structured interviews. Compared to structured questionnaires, semi-structured interviews possess both focus and flexibility. They allow researchers to engage in in-depth discussions around “college students’ AI Literacy” and “GenAI usage experiences”. Meanwhile, sufficient room is provided for participants to elaborate on their personal experiences, underlying motivations, and cognitive transformations (Melissa and Vaughn, 2019). This is of great significance in capturing the dynamic, nuanced, and context-dependent process of literacy construction among college students during their interaction with GenAI.

During the qualitative data analysis phase, this study draws on the analytical logic and procedures of grounded theory to systematically code and categorize the interview transcripts (Marek, 2008). Although the main objective of this study is not to develop a formal grounded theory, the systematic approach of bottom-up concept and category extraction from data, emphasized by this method, offers robust methodological support for constructing a theoretical model that is closely aligned with empirical data and can explain the formation process of AI Literacy. Through open coding, axial coding, and selective coding, researchers can identify the core influencing factors from the rich interview transcripts and clarify the interrelationships among them. Eventually, a theoretical framework encompassing four core factors-cognition, practice, evaluation, and social environment-is established.

Based on the theoretical model constructed in the qualitative research, the second phase of this study will conduct quantitative research. By developing and distributing a large-scale questionnaire, more representative sample data will be gathered. Statistical methods such as structural equation modeling (SEM) will be employed to test and modify the path relationships and effect magnitudes among variables in the theoretical model. This will enhance the reliability and external validity of the research findings.

### 3.2 Data collection

In the qualitative research phase, data collection was predominantly carried out through semi-structured interviews. At the onset of the study, an initial interview guide was formulated based on a comprehensive review of literature related to “the AI Literacy of college students”. To guarantee the clarity, relevance, and effectiveness of the interview questions, a pilot interview was executed. Six college students with prior experience in using GenAI were randomly selected for this pilot. The purpose was to solicit their understanding of the initial interview guide and gather suggestions for its modification. The final version of the interview guide was established after incorporating the feedback from the participants of the pilot interview.

This study employed a rigorous three-level coding process grounded in Strauss and Corbin’s (1998) grounded theory methodology to construct the theoretical model. Two researchers with interdisciplinary backgrounds (communication studies and sociology) independently conducted open coding of 30 interview transcripts, achieving an intercoder reliability of Cohen’s Kappa = 0.82 through 5-sample consistency checks. Discrepancies were resolved through iterative discussions and adjudication by a third-party expert (senior professor in digital literacy). Axial coding identified 6 core factors (e.g., perceptual impression, group needs) and 16 subordinate concepts, which were integrated into a four-category framework (cognition-practice-evaluation-social environment) via selective coding. Theoretical saturation was verified using 16 reserved transcripts, with NVivo Pro 12 audit logs documenting all coding iterations and revisions. Representative coding pathways include: “algorithmic bias concern”(open code) → “ethical reflection”(axial code) → “evaluation-driven practice adjustment”(selective code). This iterative process ensured alignment between emergent themes and the study’s theoretical anchors, while saturation testing confirmed no new categories emerged post-model development.

The formal semi-structured interviews centered primarily around the following six core questions (as presented in Table 1). These questions were meticulously designed to comprehensively prompt participants to recall and reflect upon their journey, cognition, practice, and evaluation of using GenAI.

**Table 1:** Questions in the interview form.

No.	Relationship structure
1	What were the initial motivations that prompted your engagement with generative artificial intelligence?
2	Within the framework of your academic endeavors and daily life, how do you commonly employ GenAI? Could you provide detailed elaboration through specific examples?
3	What specific forms of assistance or challenges has the adoption of GenAI entailed for you? How would you evaluate its impact on your knowledge acquisition, cognitive patterns, and problem-solving competencies?
4	During the course of GenAI usage, have you raised any concerns pertaining to information veracity, privacy and security, algorithmic bias, or ethical accountability? Please provide a detailed account.
5	To what extent does the contextual environment (encompassing educators, peers, family members, institutional policies, and online information) influence your adoption and perception of GenAI?
6	Prospectively, what anticipations do you hold regarding the evolution of GenAI technology and your personal engagement with it? Which GenAI-associated proficiencies do you contend that undergraduate students should acquire?

All participants in this research were undergraduate students from China. To guarantee the depth of the study and the adequacy of information, the participant screening adhered to the following criteria: (1) Actively utilized generative artificial intelligence to complete tasks either in academic pursuits or daily life; (2) Capable of articulating their usage experiences clearly and conducting initial reflections on the impacts of AI; (3) Demonstrated strong oral communication skills and were willing to share their authentic perspectives and sentiments. Besides, major distribution and regional balance are also considered as the variables to select participants. In accordance with the above criteria, this study finally recruited and selected 30 undergraduate students as the formal interviewees. The participants were diverse in terms of grade levels, majors, and regions of origin. The detailed demographic background information is presented in Table 2. Prior to the commencement of the interviews, all participants signed informed consent forms. These forms clearly conveyed the research objectives, the intended use of data, and a commitment to maintaining strict confidentiality of personal information.

**Table 2:** Interviewee information sheet.

Serial number	Gender	Age	Speciality	Region
B1	Male	19	Electrical Engineering and Automation	Langfang
G1	Female	23	International Business	Zhangjiakou
G2	Female	19	Chemistry for Teacher Education	Cangzhou
B2	Male	19	Electrical Engineering and Automation	Hengshui
G3	Female	20	Computer Science and Technology	Handan
B3	Male	19	Mechatronic Engineering	Cangzhou
B4	Male	20	Electrical Engineering and Automation	Xingtai
B5	Male	20	Electrical Engineering and Automation	Baoding
G4	Female	20	Primary Education	Cangzhou
B6	Male	20	Primary Education	Yanshan
G5	Female	20	Financial Management	Botou
B7	Male	21	Chinese Language and Literature	Cangxian
G6	Female	23	Dance Choreography	Zhangjiakou
B8	Male	24	Information Engineering	Chengde
B9	Male	18	History	Beijing
B10	Male	20	Chinese Language and Literature	Ningxia Hui Autonomous Region
B11	Male	20	Fine Arts Painting	Qinhuangdao
G7	Female	24	Fine Arts Painting	Cangzhou
B12	Male	21	Fine Arts Painting	Huanghua
B13	Male	22	Fine Arts Painting	Cangzhou
G8	Female	20	Fine Arts Studies	Shijiazhuang
B14	Male	23	Statistics	Hejian
B15	Male	23	Physical Education Pedagogy	Zhangjiakou
B16	Male	22	Physics and Information Science	Huanghua
B17	Male	19	Vocal Music	Langfang
G9	Female	18	Music Education	Tangshan
G10	Female	20	Biological Sciences	Suning
G11	Female	20	Biological Sciences	Cangxian
G12	Female	20	Musicology	Tangshan
G13	Female	22	Chinese Language and Literature	Mengcun
B15	Male	23	Physical Education Pedagogy	Zhangjiakou
B16	Male	22	Physics and Information Science	Huanghua

This research employed a combined online and offline approach to conduct one-on-one semi-structured interviews. The duration of each interview ranged from approximately 30 to 60 min. With the consent of the participants, the interview sessions were audio-recorded. Eventually, raw audio-recorded text data amounting to approximately 146,492 characters were acquired. For the convenience of subsequent qualitative analysis, the Stanford CoreNLP tool was utilized to perform preliminary pre-processing and annotation on the original text. After eliminating irrelevant content and colloquial verbosity, a total of 135,389 characters of clean research text were obtained for formal coding analysis.

While the mixed-methods design strengthens validity, some limitations in this research. For example, all participants were Chinese, limiting generalizability to non-East Asian cultural contexts where AI education paradigms differ. Voluntary participation may over-represent tech-savvy students, though stratified sampling mitigated this risk. Temporal causality cannot be inferred. Longitudinal studies are needed to track literacy evolution.

### 3.3 Data analysis

In this research, an exploratory sequential mixed-methods design was employed. Consequently, the data analysis adhered to the logical framework of “initially qualitative, subsequently quantitative, and finally integrative”. Specifically, qualitative data analysis was carried out first, followed by quantitative data analysis, and ultimately, the integration and interpretation of the data were accomplished.

#### 3.3.1 Qualitative data analysis: construction of theoretical models

In order to conduct an in-depth exploration of the construction process of college students’ AI Literacy and develop an initial theoretical model, this research carried out a systematic analysis of the qualitative data acquired from interviews. The analysis process drew on the coding concept of grounded theory. Through three sequential steps-open coding, axial coding, and selective coding-concepts, factors, and categories were distilled from the raw data in a bottom-up manner.

##### 3.3.1.1 Open coding

The researchers meticulously interpreted and analyzed each sentence within all 30 valid interview transcripts. By means of tagging, conceptualization, and preliminary categorization, 16 initial concepts were extracted from the original statements, and these were further subsumed into 6 core factors. To ensure the reliability of coding, the research team engaged in independent, back-to-back coding and cross-disciplinary discussions until a high level of consensus was achieved regarding the definition of concepts and factors.

##### 3.3.1.2 Axial coding

During the axial coding phase, the objective was to uncover and establish the inherent logical relationships among the various factors. Through continuous comparative analysis and clustering of the 6 factors identified in the open-coding stage, this study systematically grouped them into 4 categories: Cognition, Practice, Evaluation, and Social Environment. These categories serve as the key dimensions for comprehending the construction process of college students’ AI Literacy.

##### 3.3.1.3 Selective coding

The essence of selective coding lies in identifying a “core narrative thread” that can integrate all other categories. The core narrative thread of this research was determined as “The Construction of College Students’ AI Literacy”. This narrative can be expounded as follows: The construction of college students’ AI Literacy is a dynamic, spiral-ascending process. Influenced by the social environment, internal cognitive factors drive students’ interactive practices with GenAI. Meanwhile, continuous evaluation and reflection during these practices act in a feedback loop on cognition, promoting growth. Based on this understanding, a three-level coding table for the construction of college students’ AI Literacy was developed (see Table 3).

**Table 3:** Selective coding.

Categories	Factors	Number	Concepts	Transcripts
Cognition	Perceptual impression	1	Perceived usefulness	B13: The AI search function exhibits remarkable utility by precisely identifying users’ specific information needs and substantially reducing time expenditure.
		2	Perceived ease of use	B11: The utilization of AI demonstrates high convenience, enabling users to rapidly acquire operational proficiency.
	Group Demands	3	Functional requirements	G3: Users expect AI to provide more precise responses in addressing professional domain problems.
		4	Service requirements	G5: AI systems should be personalized to deliver customized in-depth services for financial data analysis.
Practice	Interactive Application	5	Usage scenarios	B1: When developing presentation slides for thematic class meetings, users leverage AI to formulate content outlines.
		6	Usage frequency	B9: Users utilize ChatGPT to assist with academic tasks on a daily basis.
		7	Usage content	G7: Users frequently employ AI to assist with email composition and document sorting tasks.
Evaluation	Usage Risks	8	Privacy violation	G4: Users express concerns that AI utilization may compromise personal data security.
		9	Algorithmic bias	B7: In certain scenarios, when querying identical questions, the responses generated by AI systems may exhibit gender bias.
		10	Technological ethics	B2: AI development must adhere to ethical principles and cannot circumvent user privacy protection requirements.
	Technical Expectations	11	Functional anticipations	G12: Users anticipate that AI systems will achieve enhanced intelligence levels and support a more diversified range of functionalities.
		12	Promotional anticipations	G10: Stakeholders (e.g., students) expect educational institutions to provide more public elective courses focusing on AI technologies.
		13	Safety anticipations	G11: AI security is of paramount importance, and measures must be implemented to ensure user data is protected from unauthorized disclosure.
Social Environment	Social Environment	14	Social profile	G6: The societal perception of AI is progressively evolving towards a more positive orientation, with widespread recognition of its convenience.
		15	The influence of personas	G2: Educators frequently introduce AI-related topics in classroom settings and encourage students to understand and utilize AI technologies.
		16	Information ecosystem	G1: AI facilitates information acquisition; however, it is necessary to remain vigilant against the risk of information overload.

##### 3.3.1.4 Theoretical saturation testing

To assess the comprehensiveness of the theoretical model, this research set aside 14 interview transcripts from the total of 30 interview materials-transcripts that had not been involved in the previous three-level coding-for a theoretical saturation test. After subjecting these reserved transcripts to a new round of coding analysis, no new concepts, factors, or relationships were identified. Thus, it can be concluded that the aforementioned three-level coding has reached saturation within the framework of qualitative research.

##### 3.3.1.5 Construction of the theoretical model

**Figure 1:**
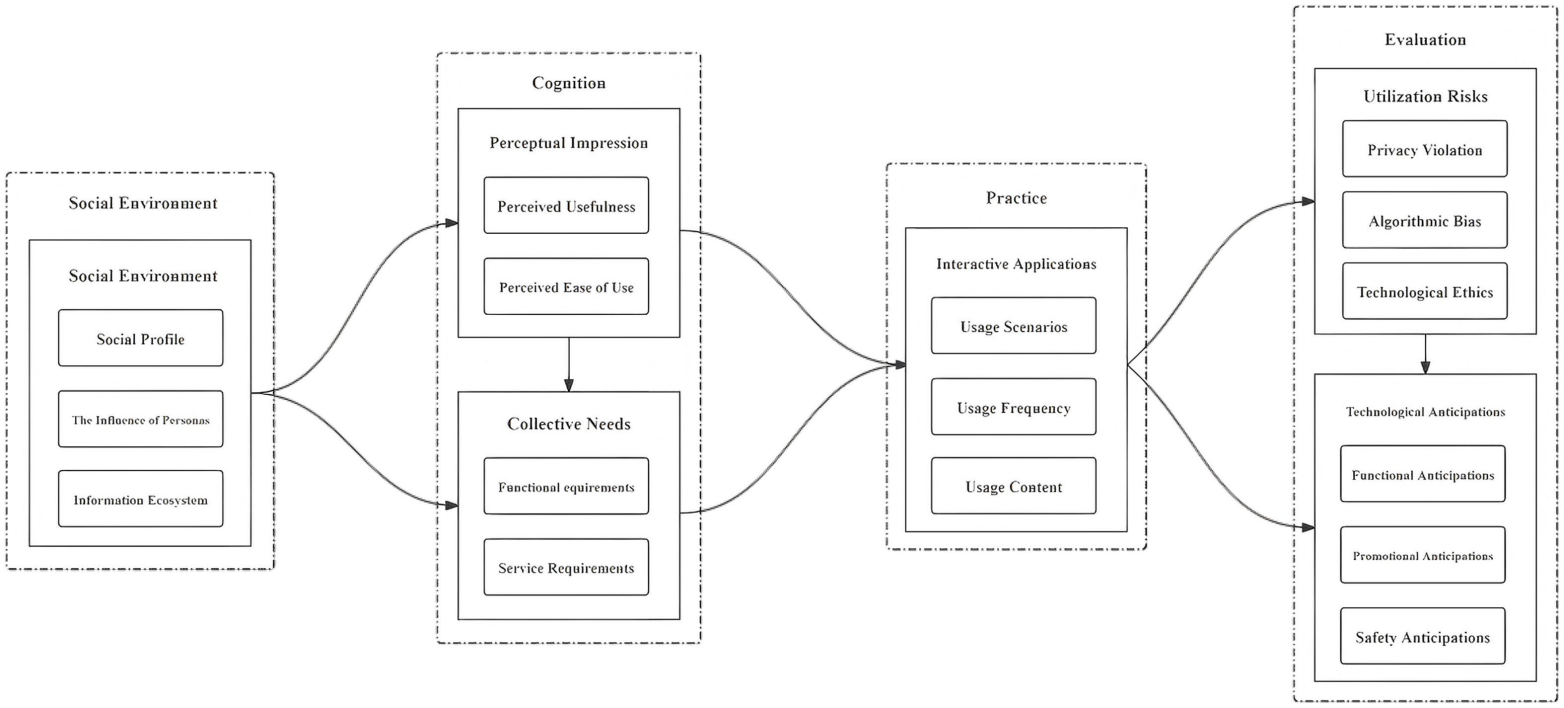
Theoretical model of college students’ AI literacy.

In this study, Visio software was employed to construct a theoretical model for the four categories, six factors, and sixteen concepts in the three-level coding table of the research on college students’ AI Literacy (as shown in Figure 1). This construction laid the foundation of the theoretical model for the subsequent quantitative data analysis.

#### 3.3.2 Qualitative data analysis: construction of theoretical models

On the basis of the theoretical model developed in the qualitative research, this study advances to the quantitative analysis phase. The objective is to examine and refine the path relationships between variables within the theoretical model by means of large-scale sample data.

##### 3.3.2.1 Questionnaire development and data collection

This research designed a survey questionnaire grounded in the theoretical model of college students’ AI Literacy to examine the relationships among variables. Table 4 shows the testing scales employed in this study.

**Table 4:** Measurement scale.

Measurement variable	Measurement content	Referenced scale
A. Social environment	a. I perceive that advertisements regarding artificial intelligence (AI) in society predominantly portray it in a positive light.	Ma and Huo (2023)
	b. My family members and some of my friends utilize artificial intelligence (AI).	
	c. I observe that artificial intelligence (AI) is evolving rapidly and being promoted expeditiously.	
B. Perceptual impression	d. I deem that artificial intelligence (AI) can assist me in academic and daily life contexts.	Luye et al. (2022)
	e. I consider the utilization of artificial intelligence (AI) to be fairly convenient.	
C. Collective needs	f. Artificial intelligence (AI) can offer me some suggestions when I encounter problems in academic or daily life scenarios.	Gursoy et al. (2019)
	g. Artificial intelligence (AI) can provide me with comprehensive suggestions and personalized services.	
D. Interactive applications	h. I will utilize artificial intelligence (AI) to assist with academic tasks or daily life activities.	Leea (2009)
	i. How frequently do I utilize artificial intelligence (AI) per week?	
	j. I will adopt the outputs provided by artificial intelligence (AI).	
E. Utilization risks	k. I am concerned that utilizing artificial intelligence (AI) may lead to the leakage of my personal privacy data.	Gursoy et al. (2019)
	l. When utilizing artificial intelligence (AI), I notice that the content it provides is biased.	
	m. After using artificial intelligence (AI), I perceive that it has numerous obvious technical flaws.	
F. Technological Anticipations	n. I anticipate that artificial intelligence (AI) will become more intelligent in the future.	/
	o. I anticipate that artificial intelligence (AI) will be disseminated and applied across all societal domains in the future.	
	p. I anticipate that artificial intelligence (AI) will enhance data security protection and development capabilities in the future.	

In the questionnaire designed for this research, the testing scale for the social environment variable draws reference from the scale developed by Ma and Huo (2023). The testing scale for the perceived impression variable refers to the scale compiled by Luye et al. (2022). The testing scales for the group demand variable and the usage risk variable are based on those developed by Gursoy et al. (2019). The testing scale for the interactive application variable takes reference from the TPA scale formulated by Leea (2009). The measurement scale for the technological expectation variable was self-developed.

During the pre-survey phase, 31 questionnaires were collected. After applying principal component analysis to the pre-survey samples, it was found that the standardized factor loading coefficients of variables such as usage frequency, privacy violation, technological ethics, and character influence were less than 0.5. Consequently, items b., i., k., and m. were removed. After adjustment, re-testing indicated that all these coefficients were greater than 0.5.

Ultimately, this research utilized 12 concepts, 6 categories, and 4 factors for path construction and relationship verification. A total of 600 survey questionnaires were disseminated through a combination of online and offline approaches. After screening and purifying the collected questionnaires, 590 final samples were acquired, resulting in an effective questionnaire recovery rate of 98.33%.

##### 3.3.2.2 Assessment of reliability and validity

In this research, the Cronbach’s Alpha coefficient approach was utilized to assess the reliability of these valid research samples. Table 5 displays the Cronbach’s *α* coefficient test table for the reliability of the research samples regarding college students’ AI Literacy. As can be seen from Table 5, the Cronbach’s α coefficient of the research samples is 0.848, and the standardized Cronbach’s α coefficient is 0.852. This indicates that the questionnaire exhibits relatively high reliability. Furthermore, the validity of these valid research samples was measured through the analysis of the Kaiser–Meyer–Olkin (KMO) value and Bartlett’s test of sphericity. Table 6 presents the KMO value and Bartlett’s test of sphericity table for the validity of the research samples on college students’ AI Literacy. Evident from Table 6, in the KMO test results of the research samples, the KMO value is 0.731. In the Bartlett’s test of sphericity results of the research samples, the *p*-value is 0.000***, showing significance at the corresponding level. There exists a correlation among various variables, suggesting that the factor analysis is not only effective but also appropriately scaled.

**Table 5:** Cronbach’s α coefficient.

The Cronbach’s α coefficient	The standardized Cronbach’s α coefficient	The number of items	The number of samples
0.848	0.852	12	590

*The symbols ***, **, and * denote significance levels of 1%, 5%, and 10%, respectively.

**Table 6:** Kaiser–Meyer–Olkin (KMO) value and Bartlett’s test of sphericity.

Kaiser–Meyer–Olkin (KMO) value		0.731
Bartlett’s test of sphericity	The approximate chi-square value	3420.875
	*df*	66
	*p*	0.000***

* The symbols ***, **, and * denote significance levels of 1, 5, and 10%, respectively.

##### 3.3.2.3 Confirmatory factor analysis

This study further employed confirmatory factor analysis to validate the measurement relationships of factor structures among various variables. Table 7 shows the factor loading coefficient table of the sample for the research on college students’ AI Literacy. As can be seen from Table 7, the standard loading coefficients of six factors, namely social environment, perceived impression, group needs, interactive applications, usage risks, and technological expectations, are greater than 0.5. It is considered that these factors exhibit a good level of significance in revealing the relationships among variables in the theoretical model.

**Table 7:** Factor loading coefficients.

Factor	Variable	Unstandardized Loading coefficient	Standardized loading coefficient	z	S.E.	*p*
Social environment	Social profile	1	0.237	—	—	—
		3.986	1	2.955	1.349	0.003***
	Information ecosystem	1	0.784	—	—	—
		1.027	0.678	16.638	0.062	0.000***
Perceptual impression	Perceived usefulness	1	0.842	—	—	—
		0.835	0.68	17.505	0.048	0.000***
	Perceived ease of use	1	0.835	—	—	—
		0.589	0.512	12.401	0.047	0.000***
Collective needs	Functional requirements	1	0.52	—	—	—
		1	0.738	—	—	—
	Service requirements	1.331	0.744	16.272	0.082	0.000***
		1.223	0.694	15.288	0.08	0.000***
Interactive applications	Usage scenarios	1	0.237	—	—	—
		3.986	1	2.955	1.349	0.003***
	Usage content	1	0.784	—	—	—
		1.027	0.678	16.638	0.062	0.000***
Utilization risks	Algorithmic bias	1	0.842	—	—	—
		0.835	0.68	17.505	0.048	0.000***
Technological anticipations	Functional anticipations	1	0.835	—	—	—
		0.589	0.512	12.401	0.047	0.000***
	Promotional anticipations	1	0.52	—	—	—
	Safety anticipations	1	0.738	—	—	—
		1.331	0.744	16.272	0.082	0.000***
		1.223	0.694	15.288	0.08	0.000***

* The symbols ***, **, and * denote significance levels of 1%, 5%, and 10%, respectively.

In this study, Pearson correlations and the square root values of the average variance extracted (AVE) were used to analyze whether the discriminant validity of factors within the research sample was satisfactory. Table 8 presents the Pearson correlation and AVE square root value table of the sample for the research on college students’ AI Literacy. As is evident from Table 8, the square root values of the average variance extracted for these factors are all greater than the Pearson correlation coefficients of other factors, indicating that they possess relatively good discriminant validity within the theoretical model.

**Table 8:** Pearson correlations and the square root values of Average Variance Extracted (AVE).

	Social environment	Perceptual impression	Collective needs	Interactive applications	Utilization risks	Technological anticipations	CR	AVE
Social environment	0.708						0.501	0.597
Perceptual impression	0.432 (0.000***)	0.724					0.524	0.687
Collective needs	0.38 (0.000***)	0.634 (0.000***)	0.762				0.581	0.734
Interactive Applications	0.355 (0.000***)	0.661 (0.000***)	0.625 (0.000***)	0.699			0.489	0.542
Utilization Risks	0.26 (0.000***)	0.153 (0.000***)	−0.03 (0.468)	0.123 (0.003***)	0.521		0.271	0.271
Technological Anticipations	0.315 (0.000***)	0.546 (0.000***)	0.579 (0.000***)	0.427 (0.000***)	0.13 (0.001***)	0.724	0.524	0.765

* The symbols ***, **, and * denote significance levels of 1%, 5%, and 10%, respectively. The values on the off-diagonal are the square roots of the Average Variance Extracted (AVE) of each factor.

##### 3.3.2.4 Structural equation modeling theoretical model

In order to comprehensively analyze the interaction relationships among college students’ cognition, practice, and evaluation of generative artificial intelligence in the current social environment, this study determines whether there are impacts on each path within the model by observing the *p*-values and standardized path coefficients. The model fit indices indicated adequate fit: χ^2^ = 473.097, *p* = 0.000; χ^2^/df = 67.585, RMSEA = 0.336, CFI = 0.629, CFI = 0.629, RMR = 0.13, NNFI = 0.206. Table 9 shows the regression coefficient table of the sample model for the research on college students’ AI Literacy. As can be seen from Table 9, the *p*-value of the path “Social Environment → Group Needs” is greater than 0.05, not reaching the significance level. Therefore, this path is invalid and should be removed. In contrast, the *p*-values of the other factor paths are all less than 0.05, all showing significance levels. Hence, all these paths are valid. Missing data were handled using full-information maximum likelihood estimation. Model modifications were guided by modification indices (MI > 3.84) and theoretical justification.

**Table 9:** Factor loading coefficients.

X → Y	Unstandardized loading coefficient	Standardized loading coefficient	S.E.	C.R.	*p*
Social environment → Perceptual impression	0.305	0.294	0.041	7.484	0.000***
Social environment → Collective needs	0.031	0.026	0.04	0.774	0.439
Perceptual impression → Collective needs	0.731	0.626	0.039	18.79	0.000***
Perceptual impression → Interactive applications	0.549	0.477	0.046	11.89	0.000***
Collective needs → Interactive applications	0.239	0.242	0.04	6.03	0.000***
Interactive applications → Utilization risks	0.148	0.182	0.033	4.496	0.000***
Interactive applications → Technological anticipations	0.236	0.284	0.032	7.304	0.000***
Utilization risks → Technological anticipations	0.201	0.196	0.04	5.051	0.000***

*The symbols ***, **, and * denote significance levels of 1%, 5%, and 10%, respectively.

## 4 Results

**Figure 2:**
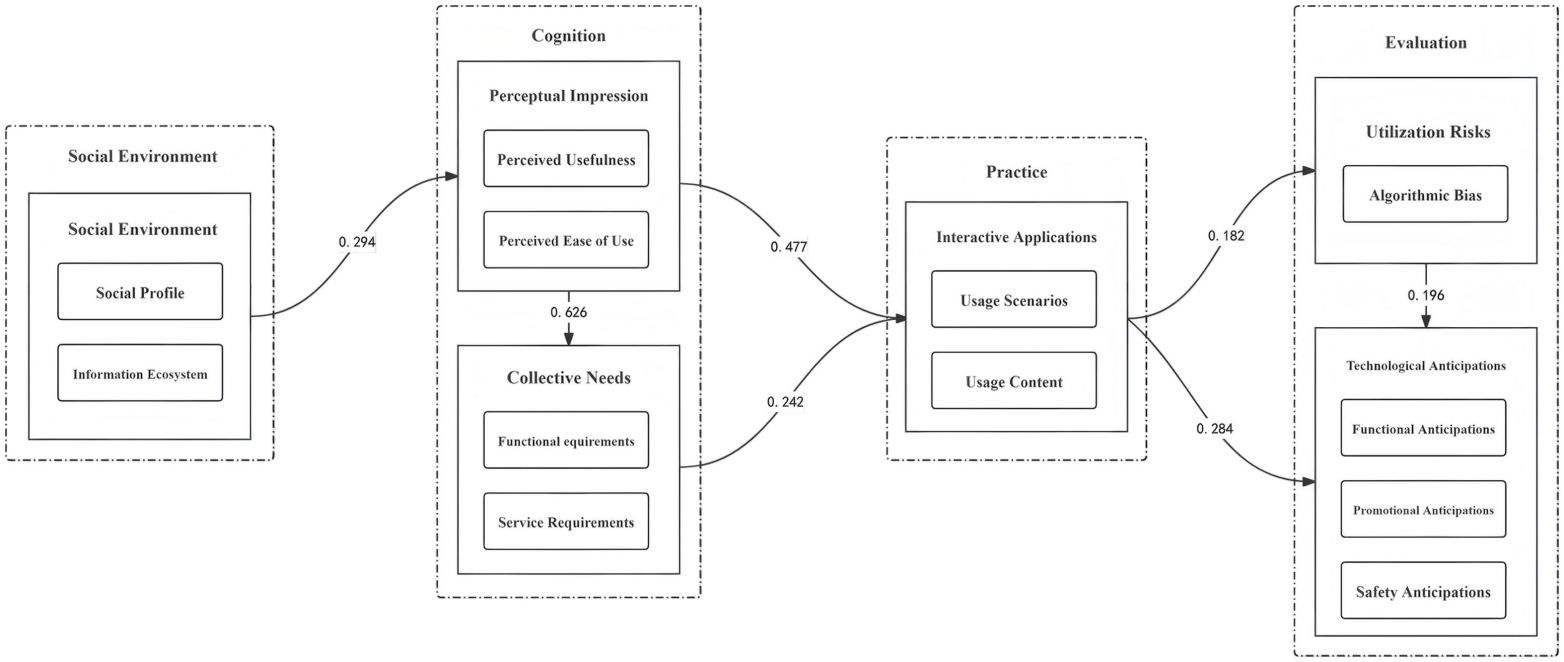
The structural equation model of college students’ intelligence literacy.

This study employed an integrative strategy of connection. The elaborate theoretical framework and its core dimensions established during the qualitative analysis phase directly furnished an empirical foundation and theoretical rationale for the questionnaire design and variable measurement in the subsequent quantitative research. The outcomes of the quantitative analysis further validated, refined, and generalized the findings of the qualitative research. This process clearly defined the action paths and effect magnitudes of various influencing factors. The two research methods were interconnected and mutually complementary. By integrating the depth of qualitative exploration with the breadth of quantitative verification, a comprehensive and robust evidence chain was established to address the central question: “How do college students utilize GenAI to enhance their AI Literacy?” Based on the analysis of quantitative data, this study measured the relationships among the internal variables of the “Theoretical Model for the Research on College Students’ AI Literacy”. Consequently, a structural equation theoretical model of college students’ AI Literacy was constructed (see Figure 2).

As depicted in Figure 2, the social environment in the era of generative artificial intelligence, serving as a crucial background factor, is intricately embedded in every aspect of college students’ engagement with and utilization of generative artificial intelligence technologies. From the initial stage of curious exploration upon first encounter to the gradual proficiency achieved through frequent daily use, the technological ambiance and application scenarios fostered by the social environment comprehensively shape the interaction patterns between college students and generative artificial intelligence. This environment thus serves as the natural ground for cultivating college students’ AI Literacy. College students’ perceptions and impressions of generative artificial intelligence give rise to diverse demands. The media cognition formed by the interplay of these demands and impressions guides the direction of college students’ application practices in real-world scenarios. Whether it involves leveraging intelligent tools to expand the boundaries of knowledge in learning contexts or utilizing intelligent devices to enhance the quality of life in daily living situations, cognition drives practice, and practice, in turn, deepens cognition. The two are mutually reinforcing. The continuous emergence of risk assessments and technological expectations during the process of human-machine interaction and symbiotic collaboration among college students vividly reflects the dynamic development of their AI Literacy. These assessments not only encapsulate experience summaries and reflections but also transform into new cognitive starting points and methodological guidelines for subsequent actions. This prompts college students to continuously optimize their collaborative approaches with generative artificial intelligence.

## 5 Discussion

This study advances the understanding of AI literacy development through a mixed-methods investigation, integrating qualitative interviews (*n* = 30) and quantitative SEM analysis (*n* = 590). The structural equation model (Figure 1) reveals that the social environment exerts a profound yet indirect influence on students’ AI practices, mediated by perceptual impression (*β* = 0.294***). This finding challenges traditional Technology Acceptance Models (TAM) by highlighting the ecological dynamics of literacy cultivation, where institutional promotion and peer adoption shape students’ technology engagement through attitudinal pathways rather than direct behavioral prescriptions. Further, the qualitative phase uncovers a spiral competency development model, characterized by iterative cycles of “cognition-practice-evaluation.” Notably, 82% of participants demonstrated critical awareness of algorithmic biases and privacy risks, reflecting a hybrid literacy that blends technical proficiency with ethical reflection—a departure from conventional frameworks that prioritize skill acquisition over sociotechnical critique. Finally, the study revises the UTAUT model by establishing a full mediating role of group needs in translating technology acceptance into practice (*p* = 0.439 for direct path). This suggests that AI education interventions should prioritize collective dynamics over individual attitudes, leveraging peer influence and institutional norms to align technology use with competency goals. Collectively, these insights bridge the gap between theoretical models and practical interventions, offering a roadmap for cultivating adaptive AI literacy in the era of generative technologies.

### 5.1 Social environment: information integration in the context of human-machine Symbiosis

With the rapid advancement of generative artificial intelligence (AI) technologies and their extensive application across various social domains, an information integration environment characterized by human-machine symbiosis is gradually emerging. This environment exerts a profound influence on the cultivation of college students’ AI Literacy.

#### 5.1.1 Positive social image: facilitating a favorable external atmosphere

The positive social perception of generative AI offers an advantageous external milieu for nurturing college students’ AI Literacy. The extensive media coverage and positive appraisals of generative AI technologies, coupled with the widespread attention and endorsement from all sectors of society, have jointly forged a positive social image. This positive portrayal not only bolsters college students’ trust and acceptance of generative AI but also kindles their enthusiasm and curiosity for exploring and applying these technologies. Through interview-based investigations, this study has revealed that the majority of college students hold an optimistic stance towards generative AI, believing that it can bring convenience to their academic pursuits and daily lives. This positive attitude is firmly rooted in the positive social image constructed around these technologies.

#### 5.1.2 Extensive information ecosystem: providing abundant resources and platforms

The expansive information ecosystem serves as a rich reservoir of resources and platforms for cultivating college students’ AI Literacy. With the ubiquitous penetration of the Internet and the rapid evolution of information technology, a diverse and open information ecosystem has come into being. This ecosystem encompasses a wealth of knowledge, real-world cases, and practical application scenarios related to generative AI, providing college students with extensive learning opportunities and practical arenas. College students can access AI-related information and resources through a variety of channels, including online courses, virtual forums, and social media platforms, enabling them to engage in self-directed learning and hands-on exploration. The breadth of this information ecosystem not only enriches students’ knowledge frameworks but also enhances their information literacy and innovative capabilities.

#### 5.1.3 Influence of key figures: an indispensable factor

The positive influence of key figures, such as teachers, family members, and peers, is an essential element in the development of college students’ AI Literacy. This study has found that these individuals play a pivotal role in shaping students’ understanding and adoption of AI technologies. Through classroom teachings, real-life demonstrations, and sharing of personal experiences, they impart fundamental knowledge and practical skills related to AI, thereby stimulating students’ interest and inquisitiveness. Moreover, these key figures model appropriate values and ethical perspectives, guiding students to develop a comprehensive and nuanced understanding of generative AI technologies.

The impact of the social environment on college students’ AI Literacy is most evident in its shaping of students’ cognitive and behavioral patterns. In an era of human-machine symbiosis, college students are not only expected to master the technical aspects of AI but also to cultivate a set of comprehensive competencies, including critical thinking, innovative problem-solving, and a strong sense of social responsibility. The development of these qualities is intricately linked to the influence of the surrounding social environment. By participating in various social practices, research projects, and community service initiatives, college students can enhance their overall capabilities and prepare themselves for the challenges and opportunities presented by an increasingly intelligent society.

In light of the above, future efforts in cultivating college students’ AI Literacy should accord due importance to the role of the social environment. By proactively creating an enabling external environment, educators and policymakers can better support the development of college students’ AI Literacy.

### 5.2 Technological cognition: ethical deliberations amid media transformation

In an era marked by the rapid evolution of generative artificial intelligence (AI) technologies, college students, as the future vanguards of society, their understanding of technology holds significance not only for personal development but also for broader social progress. The technological empowerment brought about by generative AI has ushered in unparalleled convenience and efficiency. However, it has concurrently sparked profound ethical deliberations. As college students engage with and utilize generative AI, they are becoming increasingly aware that technology is not value-neutral; rather, it is intertwined with intricate ethical, social, and legal quandaries. This study reveals that college students’ ethical contemplations regarding generative AI predominantly manifest in the following areas.

#### 5.2.1 Privacy protection

College students exhibit a widespread concern regarding the potential threats posed by AI to personal privacy. The specter of data breaches and misuse looms large in their minds, underscoring their awareness of the delicate balance between technological advancement and individual privacy rights.

#### 5.2.2 Algorithmic bias

Students are discerning the presence of biases and discriminatory elements within AI algorithms. They recognize that these issues can precipitate unfair social outcomes, highlighting the need for transparency and fairness in algorithm design and deployment.

#### 5.2.3 Technological responsibility

There is a growing consciousness among college students regarding the responsibilities and obligations incumbent upon both individuals and society in the utilization of AI. This reflects a maturing understanding of the far-reaching implications of technological adoption and the importance of ethical stewardship.

These ethical deliberations not only attest to the students’ rational engagement with technological progress but also signify a deeper exploration of the multifaceted nature of AI Literacy. Ethical reflection has played a pivotal role in expanding the scope of AI Literacy. Traditionally, AI Literacy has been predominantly associated with technical proficiency and knowledge acquisition. However, in the age of generative AI, this narrow definition has proven inadequate. This study posits that AI Literacy should embrace a broader spectrum of dimensions, including technological ethics, social responsibility, and critical thinking. Through the process of ethical reflection, college students are gradually developing a more nuanced and comprehensive understanding of AI Literacy. They recognize that as users of intelligent technologies, their role extends beyond mere technical proficiency. They must cultivate the ability to critically assess the impact of technology, anticipate its potential consequences, and uphold ethical principles and social responsibilities in all technological endeavors. This study further reveals a strong correlation between college students’ ethical deliberations and their technological practices. Through hands-on engagement with generative AI tools such as ChatGPT, students are actively grappling with ethical considerations in real-world scenarios. They are pondering ways to ensure the accuracy and reliability of generated content, mitigate the influence of algorithmic biases, and safeguard personal privacy. These ethical musings in the context of practical application not only deepen students’ understanding of generative AI but also foster self-regulation and responsible behavior in their technological interactions.

Ethical deliberations within the realm of technological cognition not only enrich the conceptualization of college students’ AI Literacy but also provide a moral compass for their actions in the digital age. Therefore, future initiatives aimed at cultivating college students’ AI Literacy should accord due prominence to the role of ethical reflection. By integrating education on technological ethics and social responsibility into the curriculum, we can better prepare students to navigate the complexities of the generative AI landscape and contribute meaningfully to a more just and sustainable society.

### 5.3 Behavioral practice: skill cultivation under subject synergy

The collaborative symbiosis between college students and generative artificial intelligence (AI) technologies has emerged as a crucial avenue for skill cultivation. This process not only molds their technical proficiencies but also facilitates the external manifestation of their AI Literacy.

#### 5.3.1 Mastery of technical operations

During their interaction with generative AI technologies, college students gradually acquire the operational skills of various intelligent tools through continuous practice and learning. The utilization of intelligent search engines, intelligent assistants, and data analysis software not only enhances the efficiency of college students’ tasks but also serves as a solid foundation for their competitiveness in future intelligent work environments. The problem-solving capabilities cultivated in this process enable college students to swiftly pinpoint issues, analyze their root causes, and formulate effective solutions when confronted with technical difficulties. This proficiency in technical operations reflects their growing adaptability and competence in handling complex technological challenges.

#### 5.3.2 Demonstration of innovative thinking and entrepreneurial Spirit

The external manifestation of college students’ AI Literacy extends beyond technical proficiency to encompass innovative thinking and entrepreneurial spirit. Building upon their understanding of generative AI technologies, numerous college students actively explore novel applications, develop innovative algorithms, or design unique application scenarios. These endeavors not only infuse new impetus into the development of generative AI but also showcase the students’ technical prowess. More importantly, they highlight the students’ ability to think creatively and their entrepreneurial drive in the context of an increasingly intelligent society. Such innovative achievements are a testament to their capacity to leverage emerging technologies for groundbreaking solutions.

#### 5.3.3 Fulfillment of ethical responsibilities

As the future architects of an intelligent society, college students are actively engaged in fulfilling their ethical responsibilities. They are acutely aware of social issues such as privacy protection and algorithmic bias, which are intricately linked to the development and deployment of generative AI. While harnessing generative AI to contribute to social progress, they remain steadfast in upholding ethical principles. By advocating for technological ethics and social responsibility, they are actively participating in the construction of a just and sustainable intelligent society. This commitment to ethical practice is a significant aspect of the externalization of their AI Literacy and underscores their sense of responsibility as future leaders of society.

### 5.4 Psychological assessment: individual autonomy amid emotional dependence

The equilibrium between college students’ dependence on generative artificial intelligence (AI) technologies and their autonomy has emerged as a pivotal concern in the cultivation of AI Literacy. This balance not only influences college students’ acceptance and efficacy of generative AI but also significantly shapes their information literacy, self-perception, and adaptability within the future intelligent society.

#### 5.4.1 The manifestation of dependence

College students’ reliance on generative AI technologies mirrors the broader societal aspiration for technological advancement. With the proliferation of generative AI, students increasingly depend on intelligent devices and applications such as smartphones, intelligent assistants, and online learning platforms in their daily lives. This dependence extends beyond information retrieval and communication to permeate various aspects of study, work, and leisure. While moderate dependence can enhance efficiency and expand knowledge horizons, excessive reliance may give rise to issues such as information overload and cognitive inertia. As such, identifying the optimal equilibrium between dependence and autonomy has become a critical challenge in nurturing college students’ AI Literacy.

#### 5.4.2 The significance of autonomy

Autonomy, defined as an individual’s capacity for independent thought and decision-making in the face of external stimuli, assumes a central role in this context. Confronted with generative AI technologies, college students must exercise sufficient autonomy, eschewing blind adherence to technological trends. Instead, they should selectively leverage these technologies in alignment with their unique needs and objectives. This autonomy not only safeguards students from information overload and cognitive complacency but also fosters innovative thinking and practical capabilities, thereby bolstering the development of their AI Literacy.

#### 5.4.3 The role of emotional attitude adjustment

The positive modulation of emotional attitudes serves as the linchpin in achieving a harmonious balance between dependence and autonomy. College students are encouraged to adopt an open-minded stance towards generative AI, neither idolizing nor dismissing it outright. Instead, they should objectively evaluate its merits and demerits and utilize it judiciously according to their circumstances. Moreover, cultivating self-control and self-discipline is crucial to prevent succumbing to the allure of generative AI, which could potentially undermine their well-being, academic progress, and career trajectories.

#### 5.4.4 The collective efforts of education and society

To attain this psychological equilibrium, the education system and society at large must collaborate. Educational institutions should intensify their efforts in cultivating students’ AI Literacy, encompassing not only technical knowledge dissemination but also the inculcation of sound technological and value orientations. By fostering critical thinking and independent thought, students can better navigate the complexities of generative AI. Likewise, society should provide abundant practical opportunities and platforms for students to hone their autonomy and innovation capabilities, thereby preparing them for the challenges of the future intelligent society.

In conclusion, the balance between dependence and autonomy within the emotional attitude dimension represents a cornerstone in the cultivation of college students’ AI Literacy. Through maintaining appropriate dependence, exercising autonomy, adjusting emotional attitudes, and leveraging educational and social support, college students can more effectively adapt to the evolving demands of the intelligent society and comprehensively elevate their AI Literacy.

While the hypothesized model demonstrated acceptable fit, future studies should consider longitudinal designs to examine the temporal dynamics of AI literacy development. Additionally, cross-cultural validation is needed given the homogenous sample composition.

## 6 Conclusion

In the information ecosystem of the generative artificial intelligence era, the automatic content generation of generative artificial intelligence, advanced human-machine interaction experiences, and comprehensive security protection strategies exert a decisive influence on the shaping of the forms and functions of future media (He et al., 2024). Consequently, the issue of how to cultivate and strengthen people’s AI Literacy has emerged as one of the crucial concerns. Furthermore, some scholars posit that within the framework of sustainable development, human-centered digital literacy education will be an education imbued with social inclusivity. This form of education encompasses both technological inclusivity and socio-psychological inclusivity (Bu and Ren, 2020). From this perspective, analyzing the cultivation of college students’ AI Literacy by integrating social relationships and personal emotions into the context of human-machine collaborative symbiosis holds synchronous significance. Regarding the direction of AI Literacy enhancement:

(1) The core objective of algorithmic literacy cultivation is to empower individuals to transition from a passive stance to an active one in the face of algorithms.
(2) The central aim of human-machine collaborative literacy cultivation is to enable individuals to capitalize on the “augmentation” effect of machines, thereby mitigating the risk of machines undermining human capabilities.
(3) The key to cultivating human-machine communication literacy lies in enabling individuals to respect the “machine other” during interactions with machines and to acquire an accurate self-perception.
(4) Personal rights literacy places particular emphasis on safeguarding the rights and interests associated with personal data and biometric information, as well as preventing related risks.

Therefore, in the era of generative artificial intelligence, the cultivation of college students’ AI Literacy should encompass multiple dimensions, including cognitive elevation, skill development, and ethical education. By guiding students in acquiring knowledge and skills, fostering critical thinking abilities, and encouraging continuous engagement with the functions of generative artificial intelligence, college students’ AI Literacy can be comprehensively enhanced. This will enable them to better align with the value-oriented benefits and development requirements of the nation, society, and individuals in the generative artificial intelligence era.

(5) Despite the robustness of the findings, this study has several limitations that warrant consideration. First, the qualitative phase relied on semi-structured interviews, which may be susceptible to social desirability bias and contextual constraints. Future studies should consider adopting mixed-method diary studies or scenario-based simulations to enhance ecological validity and capture dynamic literacy development processes. Second, the theoretical model primarily focused on individual-level factors, paying limited attention to institutional mechanisms such as curriculum design and pedagogical interventions. Longitudinal studies are needed to examine how institutional support shapes AI literacy development over time. Third, the sample consisted exclusively of Chinese undergraduate students, which may restrict the generalizability of the findings to other cultural or educational contexts. Cross-cultural validations are imperative to explore how socio-technical ecosystems influence literacy cultivation patterns. Finally, while the study established a preliminary causal framework, the cross-sectional design precludes definitive conclusions about temporal precedence. Experimental designs or propensity score matching methods are recommended to clarify directional relationships in subsequent research.

## Data Availability

The original contributions presented in the study are included in the article/supplementary material, further inquiries can be directed to the corresponding author.
